# Myélinolyse centropontine réversible sans hyponatrémie chez un enfant atteint de leucémie aigüe lymphoblastique: à propos d’un cas

**DOI:** 10.11604/pamj.2023.44.99.29037

**Published:** 2023-02-20

**Authors:** Mohamed Hbibi, Sarra Benmiloud, Moustapha Hida

**Affiliations:** 1 Unité d’Oncologie Pédiatrique, Service de Pédiatrie, Hôpital Mère-Enfant Centre Hospitalier Universitaire Hassan II, Fès, Maroc,; 2 Université Sidi Mohamed Ben Abdellah, Faculté de Médecine et de Pharmacie de Fès, Fès, Maroc,; 3 Laboratoire de Recherche Biomédicale et Translationnelle (LRBT), Fès, Maroc

**Keywords:** Myélinolyse, hyponatrémie, chimiothérapie, cas clinique, Myelinolysis, hyponatremia, chemotherapy, case report

## Abstract

La myélinolyse centropontine est un trouble démyélinisant affectant principalement la partie centrale du pons. Elle est associée à une myélinolyse extrapontine dans certains cas. Cela est généralement dû à une correction rapide de l'hyponatrémie et à des changements rapides des conditions osmotiques. Nous rapportons le cas d'une fille de 3,5 ans avec un diagnostic de leucémie lymphoblastique aigüe qui est admis dans notre unité d'oncologie pour neutropénie fébrile avec diarrhée. Les tests de laboratoire ont montré une neutropénie légère, une anémie normocytaire normochrome. Les tests électrolytiques étaient normaux sans hyponatrémie. Elle a été traitée par des antibiotiques et Métronidazole. Cinq jours plus tard, elle a développé une quadriparésie flasque avec mutisme. La tomodensitométrie cérébrale était normale, l´étude de liquide céphalo-rachidien (LCR) était normal sans cellules leucémiques et l'examen ophtalmologique n'avait pas montré aucune anomalie. L´imagerie par résonance magnétique cérébrale avait montré des anomalies d'hypersignal dans les régions de pons. L'enfant s'est amélioré sans traitement spécifique, et une guérison clinique et complète des symptômes neurologiques était notée. Ce cas illustre la possibilité de myélinolyse dans certaines situations non liées à l´hyponatrémie comme la malignité, la chimiothérapie.

## Abstract (en)

Central pontine myelinolysis is a demyelinating disorder mainly affecting the central pons. In some cases, it is associated with extrapontine myelinolysis. It is usually caused by rapid correction of hyponatremia and osmotic shock. We here report the case of a 3.5-year-old girl diagnosed with acute lymphoblastic leukemia admitted to our Oncology Unit with neutropenic fever and diarrhea. Laboratory tests showed mild neutropenia, normochromic normocytic anemia. Electrolyte tests were normal without hyponatremia. She received antibiotic therapy with Metronidazole. Five days later, she developed flaccid quadriparesis with mutism. Computerized tomography (CT) scan was normal, cerebrospinal fluid (CSF) examination was normal (there was no evidence of leukemic cells) and ophthalmological examination did not show any abnormalities. Brain MRI found hyperintense signal in the pons. The child improved without specific treatment, and clinical and complete neurological recovery was noted. This case highlights that myelinolysis can occur under some circumstances not related with hyponatremia such as malignancy, chemotherapy.

## Introduction

La myélinolyse centropontine est un trouble neurologique démyélinisant affectant principalement le pons, dû généralement à une correction rapide de l'hyponatrémie et à des changements rapides des conditions osmotiques [1]. Elle peut s´associer dans certains cas à une myélinolyse extrapontine. Nous présentons ce cas dans le but de montrer la possibilité de la myélinolyse dans certaines situations non liées à l´hyponatrémie comme la malignité, la chimiothérapie et de renforcer la vigilance du clinicien dans l´identification des patients susceptibles de présenter une sensibilité accrue à la démyélinisation osmotique.

## Patient et observation

**Informations relatives au patient:** une fille de 3 ans et demi, suivie pour une leucémie aigüe lymphoblastique pour laquelle elle recevait sa chimiothérapie selon le protocole marocain MARALL 2006 risque standard. Elle était admise au service d´oncologie pédiatrique pour neutropénie fébrile postchimiothérapie avec une symptomatologie digestive faite de diarrhée.

**Résultats cliniques:** la patiente était fébrile à 39°C, tachycarde à 120 battements par minute. L'examen physique était normal sans foyer infectieux évident. Poids 16 kg pour une taille 96 cm. La pression artérielle était à 110/80 mm Hg.

**Démarche diagnostique:** sur le plan biologique, elle avait une neutropénie sévère à 160 éléments /mm^3^ à l´hémogramme avec une anémie normocytaire normochrome, un syndrome infectieux avec une CRP à 120 mg/l. La fonction rénale était normale. La copro-parasitologie des selles ne montrait pas d´agent pathogène. Les tests électrolytiques étaient normaux sans hyponatrémie.

**Intervention thérapeutique:** la patiente avait reçu une antibiothérapie intra veineuse, faite d'une triple association (Céftriaxone, Gentamycine, Métronidazole) avec une ration de base faite de sérum glucosé avec des électrolytes.

**Figure 1:**
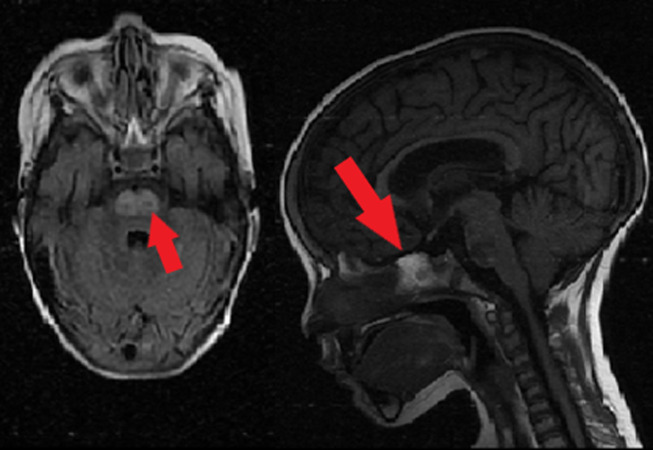
IRM cérébrale montrant une plage centropontine (flèche) en hyposignal (coupe axiale) et hypersignal (coupe sagittale)

**Suivi et résultats des interventions thérapeutiques:** l´enfant avait une apyrexie après 48 heures d´antibiothérapie. Mais cinq jours plus tard, elle avait développé une quadriparésie flasque avec mutisme. La tomodensitométrie cérébrale était normale. Dans le cadre du bilan de rechute leucémique éventuelle, les résultats du liquide céphalo-rachidien (LCR) ne montraient pas de cellules néoplasiques et l'examen ophtalmologique ne montrait aucune anomalie. Cependant, l'imagerie par résonance magnétique cérébrale avait montré des anomalies d'hypersignal dans les régions protubérance (Figure 1). L'enfant s'est amélioré sans traitement spécifique, et une guérison clinique et complète des symptômes neurologiques était notée.

**Perspectives du patient:** après la fin du traitement, la maman de l´enfant était ravie des soins qu'elle a reçus et parait optimiste quant à l'évolution de l´état de sa fille.

## Discussion

La myélinolyse centropontine (MCP) appelée aussi syndrome de démyélinisation osmotique est un processus aigu de démyélinisation impliquant le pons et d´autres localisations du système nerveux central tout en épargnant les neurones et les axones. Elle a été décrite en 1959 par Adams et ses collègues comme une maladie qui touche les patients alcooliques et dénutris [1]. Le mécanisme étiopathogénique de la MCP est non encore élucidé et semble être multifactoriel [2]. Il a été suggéré qu´il correspondrait à des lésions endothéliales osmotiques avec libération de facteurs détruisant la myéline [3] et constitution d´un œdème vasogénique. L´installation de la MCP est souvent iatrogène par une correction trop rapide d´une hyponatrémie. Dans notre cas, l´enfant n´avait pas d´hyponatrémie mais il avait une leucémie lymphoblastique et était sous chimiothérapie.

Peu de cas ont été publiés dans le même contexte. Douira-Khomsi *et al*. avaient décrit un cas de MCP chez un enfant sous chimiothérapie pour leucémie aigüe lymphoblastique avec notion d´hyponatrémie [4] tandis que Yilmaz *et al*. avaient décrit un cas de MCP chez un enfant ayant une leucémie aigüe myéloïde avec notion d´hyponatrémie et hypophosphatémie [5]. Ceci suggère qu´existe d´autres facteurs de risque de démyélinisation osmotique et de manière similaires chez les patients présentant ou non une hyponatrémie et que ces facteurs sont généralement liés à la capacité limitée de la réponse osmotique cérébrale, tels que la malignité, malnutrition, sepsis, insuffisance surrénalienne...[6]. Chez l´adulte certaines études ont montré l´implication de la chimiothérapie dans la démyélinisation osmotique en l´absence de l´hyponatrémie. En effet Jae Heun Chung *et al*. ont publié un cas de démyélinisation osmotique sans hyponatrémie après chimiothérapie à base de cisplatin [7].

Le diagnostic clinique de cette affection est généralement clinique, mais la symptomatologie est très variable selon l´étendue des lésions et son extension en extrapontine ce qui donne plusieurs formes cliniques allant de l´asymptomatique jusqu´un véritable coma. Pour la MCP, le diagnostic est évoqué devant un syndrome pseudobulbaire avec dysarthrie et mutisme, parfois même un locked-in syndrome. Des signes extrapyramidaux se voient en cas de myélinolyse extrapontine. L´imagerie par resonance magnétique (IRM) est l´examen de référence en matière de la myélinolyse osmotique. Elle montre une plage centropontine en hyposignal T1 et en hypersignal T2, FLAIR et diffusion. Les anomalies radiologiques sont habituellement retardées de l'ordre de 10 à 15 jours après l´installation des signes cliniques [8].

Le pronostic a longtemps été considéré comme grave car le diagnostic était exclusivement autopsique, cependant centaines des survivants et des récupérations neurologiques ont été décrite dans la littérature. Le traitement consiste en général en un traitement symptomatique mais essentiellement préventif reposant sur la correction progressive des hyponatrémies profondes [8].

## Conclusion

La MCP constitue une affection sévère qui peut engager le pronostic vital avec une lourde morbidité. Sa possibilité dans certaines situations non liées à l´hyponatrémie comme la malignité, la chimiothérapie, doit être présente à l´esprit du clinicien pour renforcer sa vigilance dans l´identification des patients susceptibles d´avoir une sensibilité accrue à la démyélinisation osmotique.
